# Psychosocial factors associated with the self-reported frequency of cell phone use while driving in Iran

**DOI:** 10.1371/journal.pone.0249827

**Published:** 2021-04-21

**Authors:** Amir Hossein Kalantari, Seyedeh Monavar Yazdi, Tetiana Hill, Abolfazl Mohammadzadeh Moghaddam, Esmaeel Ayati, Mark J. M. Sullman

**Affiliations:** 1 Institute for Transport Studies, University of Leeds, Leeds, United Kingdom; 2 Department of Psychology, Alzahra University, Tehran, Iran; 3 Hertfordshire Business School, University of Hertfordshire, Hatfield, United Kingdom; 4 Department of Civil Engineering, Ferdowsi University of Mashhad, Mashhad, Iran; 5 Techno-Economic Road Safety Research Center, Ferdowsi University of Mashhad, Mashhad, Iran; 6 Department of Social Sciences, University of Nicosia, Nicosia, Cyprus; Tongii University, CHINA

## Abstract

Cell phone use while driving is a common contributing factor in thousands of road traffic injuries every year globally. Despite extensive research investigating the risks associated with cell phone use while driving, social media campaigns to raise public awareness and a number of laws banning phone use while driving, this behaviour remains prevalent throughout the world. The current study was conducted in Iran, where road traffic injuries are the leading causes of death and disability, and where drivers continue to use their cell phones, despite legislative bans restricting this behaviour. A total of 255 drivers in the city of Mashhad (male = 66.3%; mean age = 30.73 years; *SD* = 9.89) completed either an online or a paper-based survey assessing the self-reported frequency of using a cell phone while driving. Psychosocial factors contributing to cell phone use while driving and support for legislation restricting this behaviour, as well as the Big Five personality traits, were also measured. Overall, the results showed that almost 93% of drivers use their cell phones while driving at least once a week, with 32.5% reporting they always use their cell phones while driving. Ordinal logistic regression revealed that the presence of a child passenger, age, perceived benefits and risks of using cell phones while driving, as well as the perceived ability to drive safely while using a cell phone, were strongly associated with the frequency of cell phone use while driving. As for personality traits—extraversion, agreeableness and conscientiousness significantly predicted the frequency of cell phone use in this sample of Iranian drivers.

## Introduction

Distracted driving contributes to thousands of road traffic injuries and fatalities around the world. According to estimates provided by the National Highway Traffic Safety Administration in 2018 [1], distracted driving accounts for approximately 8% of all fatal crashes and 15% of all injury crashes. Previous research also indicates that apart from affecting drivers themselves, distracted driving may cause injuries to other road users, such as cyclists and pedestrians [2, 3]. According to the World Health Organisation’s (WHO) Global Health Estimates [4] this issue is particularly problematic in low- and middle-income countries, where the number of deaths caused by road traffic injuries has been continuously growing since 2013. In Iran, which is a middle-income country, over the last two decades, the health burden of traffic accidents has increased by 60% [5]. Furthermore, in 2014 road traffic accidents (RTAs) caused 16,872 deaths (22 deaths per 100.000 people [6]), which is significantly higher than the global average (18.2/100.000 [7]). Additionally, according to the findings of the Global Burden of Disease Study 2010, RTAs were one of the leading causes of death, accounting for around 5% to 10% of all deaths in Iran [8]. Although the exact number of deaths caused by distracted driving in Iran is unknown, a study employing 1000 distracted drivers in Mashhad revealed that more than one-third had been involved in at least one at-fault accident over the last five years [9].

There are a number of factors that may cause driver distraction, which vary in terms of their complexity and demands posed to drivers’ mental workload [10]. However, based on the results of the recent systematic reviews and meta-analyses of on-road and naturalistic studies [11], as well as epidemiological studies [12], cell phone use while driving (CPWD) could be considered to be one of the leading types of distracted driving. Although handheld cell phone use is strictly prohibited in most countries of the East Mediterranean Region, including Iran, there is a growing body of research which shows that a substantial proportion of drivers continue to use a mobile phone while driving. For example, an observational study in Qatar found that almost 11.5% of 7,982 drivers were using their handheld mobile phone while driving [13]. A more recent Qatari study also found that 7.5% of the 2,011 drivers observed were using mobile phones while driving [14]. Another observational study conducted at major highways and inner intersections throughout Riyadh, Saudi Arabia, found that out of 1,700 drivers 13.8% were using handheld mobile phones [15]. In addition, a self-report study in Jordan found that out of the 394 drivers surveyed 35% reported making and 46% reported receiving five or more calls per day [16]. Of particular concern is the finding by Al Reesi et al. [17] that 92% of students reported regularly using a mobile phone while driving in a sample of 1,008 drivers.

As for Iran, a roadside observational study conducted in three “safe” Iranian communities (Kashmar, Khalilabad and Bardaskan) found that almost one quarter of drivers engaged in potentially distracting activities, and nearly 4% of them used a mobile phone, in a sample of 7,797 drivers [18]. Moreover, an observational on-road study in Mashhad found that out of 81,960 drivers 6.9% engaged in at least one secondary task and 5.27% used a mobile phone while driving [19]. In addition, a study in Isfahan found that at least 10% out of the 1,794 drivers observed used a handheld mobile phone, which is significantly higher than in other countries, such as Australia, USA and Canada [20]. This is rather worrying as a survey study in Tehran showed that 3.2% of participants reported injuring others because of texting and driving, while 2.8% reported being injured in a car accident because of texting and driving. In addition, 50.5% reported having experienced near-crashes while texting and driving [21].

Cell phone use is no longer limited to talking and texting, since the introduction of smartphones and social networking applications, the existing problems associated with cell phone use while driving have been exacerbated and new challenges raised for road safety experts, along with relevant policymakers and legislators. Extensive research has been conducted to investigate the detrimental effects of CPWD on driving performance and its association with RTAs. Previous research has shown that talking on a phone while driving can increase reaction and brake response times [22–25], and reduce drivers awareness of sudden events [25, 26]. Furthermore, reading or sending text messages can negatively impact longitudinal and lateral control [27], cause inappropriate variability in vehicle following distances and increase fluctuations in driving speed [27, 28]. The results of several naturalistic driving studies have also illustrated that cell phone tasks, such as texting and browsing, may cause drivers to glance away from the roadway for four-seconds or more, making it more difficult to avoid a crash [11, 29, 30].

Despite extensive research investigating the risks associated with CPWD, social media campaigns raising public awareness and a number of laws banning handheld phone use while driving, this behaviour remains prevalent throughout the world. It remains largely unknown what personal and social factors influence a driver’s decision to engage in this behaviour [31, 32]. Only a small body of research has attempted to explore this issue to bridge the existing knowledge gap [33], and only a couple of related studies have been conducted in Iran [21, 34].

### Psychosocial factors underlying cell phone use while driving

To explore why drivers continue to use cell phones while driving, various psychological variables have been previously investigated. For example, previous research has investigated the associations between CPWD and such variables as the level of problematic phone use [35], attitudes [36], beliefs [37], and risk perception [38]. Most of these studies report that the prevalence of CPWD is directly related to the positive attitudes drivers have towards this behaviour [39]. In addition, other variables, like apparent benefits and the perceived ability to drive safely and use a cell phone, as well as support for legislation restricting this behaviour, have also been found to be important factors contributing to CPWD. For example, [36] and [40] suggested that drivers who frequently use their cell phones while driving, also perceive that this behaviour can help them to use their time more efficiently [36, 40]. In addition, Przepiorka et al. [41] found that the perceived ability to drive safely while using a cell phone is a significant predictor of drivers’ intentions to read/send a text message. In turn, White et al. [42] reported that younger drivers, who approved of CPWD and perceived that they were able to engage in this behaviour and drive safely, had stronger intentions to use their phones while driving [42]. As for support for legislation, Sanbonmatsu et al. [33] reported that drivers who have negative attitudes towards cell phone use tend to be more supportive of legislation restricting CPWD. In addition, several studies report that drivers tend to have more negative attitudes about other drivers using cell phones while driving, than when they engage in this behaviour themselves, due to overestimating their own abilities, driving skills and traits [33, 43–45]. When investigating how drivers assessed their and other people’s driving attitudes, abilities, and behaviours, Sanbonmatsu et al. [33] also found that drivers tend to be inconsistent in what they do and what they advocate for others. The authors concluded that this appeared to stem from differences in the perceived safety risks of self versus other drivers’ use of mobile phones. It is worth mentioning that no previous research exploring the psychosocial factors underlying mobile phone use while driving has been conducted in Iran, where drivers continue to engage with their phones, despite the legislative bans. Therefore, to fill this gap, a questionnaire developed by Sanbonmatsu et al. [33], which includes the aforementioned variables, was used to investigate why Iranian drivers use a mobile phone while driving and whether they support legislation to restrict this risky behaviour.

### Personality traits and cell phone use while driving

In addition to various psychosocial variables to understand why certain drivers continue to put themselves at risk by using their phones while driving, previous research has also explored the influence of personality traits on this behaviour. For example, naturalistic driving research conducted on a sample of 43 drivers in the US explored the association between personality traits and drivers’ tendency to engage in risky secondary tasks [46]. Using the NEO Five Factor Inventory (NEO-FFI [47]), the authors found that drivers who scored higher in conscientiousness were less likely to engage in risky secondary tasks, performed fewer dangerous driving manoeuvres and overall had fewer crashes and near-crashes [47]. Sween et al. [48] found that higher emotionality, lower conscientiousness, openness to experience and honesty/humility were strongly associated with frequent cell phone use while driving. Using a Questionnaire Assessing Distracted Driving (QUADD) and a 45-item Big Five Personality test [49, 50], Parr et al. [51] found that higher scores in openness and conscientiousness were positively associated with greater reported texting frequency and overall frequency of CPWD in young drivers, while lower levels of agreeableness were positively related to less interacting with a phone while driving. As for older adult drivers, however, greater extraversion was strongly associated with more frequent cell phone use, especially for making and receiving calls. In addition, Braitman & Braitman [52] found that young drivers who score high in extraversion, were also more likely to engage in various secondary tasks while driving. Therefore, we can conclude that personality traits are related to CPWD, but no previous studies have been conducted to explore this issue in Iran.

## The current study

The current study was conducted from March to December in 2017 in Mashhad, Iran. The main objective of this study was to investigate the self-reported frequency of cell phone use while driving and a range of psychosocial factors underlying this risky behaviour. In particular, we explored whether and how the self-reported frequencies of CPWD are associated with psychosocial variables, such as perceived ability and risks associated with driving safely while using a cell phone, general attitudes toward CPWD and perceived benefits of this behaviour, as well as the level of support for legislation restricting CPWD. In addition, we explored participants’ attitudes toward other drivers’ usage of cell phones while driving, replicating a questionnaire developed by Sanbonmatsu et al. [33]. Lastly, we investigated whether the Big Five Personality characteristics can be used to predict the frequency of cell phone use in a sample of Iranian drivers.

## Method

### Participants

In total, 255 drivers completed either a paper-based or an online survey. Most participants were males (66.3%), with a mean age of 30.73 (range = 18–72, *SD* = 9.89). Participants’ driving experience was an average of 8.87 years (*SD* = 17.88) and they reported driving for an average of 16.58 hours per week (*SD* = 7.77). The vast majority of drivers had post-secondary education (73%) and were single (53.3%). The sample size was determined using Cochran’s formula, which can be presented as follows:

$$n=\frac{z^{2}p.q}{d^{2}}=\frac{1.96^{2}0.8.0.2}{0.05^{2}}=246$$ (1)

where *d* is the desired level of precision (i.e. the margin of error), *p* is the (estimated) proportion of the population which has the attribute in question, *q* is 1 –*p*. We found the *p* value based on our several comprehensive observational studies (see [7]) and eligibility criteria (see the next subsection).

### Procedure

Prior to conducting the study, ethical approval was granted by the Eqbal Lahoori Institute of Higher Education’s ethics committee. Before completing the survey, participants were asked to familiarise themselves with the information sheet, which outlined what the study was about, what was asked of them, their rights and the fact that their responses would be both confidential and anonymous. After that, they were asked by a researcher if they would agree to participate in the present study and their verbal consent was obtained. Data were collected using the convenience and snowballing sampling techniques. The survey was advertised on social networking apps (i.e. Telegram & Instagram) asking anyone who meets the eligibility criteria (i.e. aged above 18 years old, hold a current driver’s licence, own a cell phone, and had driven at least one hour in the past week) to complete the survey. The paper-based version of the survey was also distributed at universities, organisations, and other educational institutions (such as language and engineering institutes). Participants were also encouraged to pass on the link onto anyone else they knew who met the eligibility criteria.

### Materials

The survey consisted of a demographic section, a section on the psychosocial factors underlying cell phone use while driving, and a section investigating drivers’ personality traits using the NEO-FFI.

#### Demographics and frequency of cell phone use while driving

Participants were asked to report their age, sex, marital status, educational attainment, as well as information about their driving experience (e.g., licence tenure and average number of hours spent driving each week). Participants were also asked whether they had a hands-free device in the car they drove most regularly. In addition, participants completed a set of measures assessing cell phone use while driving. Firstly, they were asked to select the main purpose of using cell phone while driving (e.g., for calling, texting, using the Internet or social networks). Secondly, they were asked to indicate how often per week they “*Make a cell phone call*”, “*Answer a cell phone call*”, “*Write a text message*” “*Respond to a text message*”, “*Use the Internet*”, and “*Use the global positioning system (GPS*)” using a 5-point Likert scale (Never = 0 times per week; Rarely = < 6 times per week; Occasionally = ≤ 10 times per week; Often = ≤ 20 times per week; Always = ≥ 20 times per week). Those participants, who indicated that they use a cell phone while driving, were also asked to report the percentage of the time they are on the phone while driving. In addition, the drivers were posed two questions ‘*Whom are you most likely to call while driving*?’ and ‘*Whom are you most likely to text while driving*’ and asked to choose one answer from the suggested options (*Friends*, *Parents*, *Spouse/Fiancé*, *Children*, *Colleagues* and *Other*). Lastly, the drivers were asked whether they would use a cell phone if there was a child passenger in their vehicle.

#### Psychosocial factors underlying cell phone use while driving

This study largely replicated a questionnaire developed by Sanbonmatsu et al. [33], which was developed to explore the psychosocial factors influencing cell phone use while driving in a sample of University students in the US. A such, the original study contained questions assessing perceived ability to drive safely while using a cell phone, support for legislation restricting cell phone use and general attitudes toward using a cell phone while driving, perceived benefits and risks of using a cell phone while driving, as well as perceived costs of drinking and driving. These questions were also used to assess the differences in the perceived safety risks of self versus other drivers’ use of cell phones. The current study utilised the same questions, with the exception of perceived costs of drinking and driving, as it is not relevant to the Iranian context (i.e. due to prohibition of alcohol consumption, see [53]). Each of the questions are outlined in more detail below.

*Perceived ability to drive safely while using a cell phone*. Participants were asked to answer the following two questions *“To what extent are you capable of driving safely while using a cell phone*?*”* and *“To what extent do you consider others capable of driving safely while using a cell phone*?*”* which were answered using a 5-point Likert scale (where 1 = “not at all capable”, and 5 = “highly capable”).

*General attitude toward using a cell phone while driving*. To investigate drivers’ attitudes toward their use of cell phones while driving and towards other drivers’ use of cell phones while driving, they were asked to indicate to what extent they agree or disagree with the following statements “*I like to use a cell phone when I am driving*”, “*I feel positively about using a cell phone while driving*” and “*I feel positively about other people using a cell phone while driving*”. Responses were recorded using a 4-point Likert scale (1 = strongly disagree, 4 = strongly agree), which was also used to indicate agreement with the statements outlined in the next three sections.

*Perceived benefits of using a cell phone while driving*. One statement was used to explore drivers’ perceptions of the specific benefits of talking on a cell phone “*I benefit from using a cell phone while I drive (for example*, *it enables me to connect with friends and family and get work or other things done as well as makes driving less boring for me)”* and another statement was used to explore perceived general benefits of other drivers talking on a cell phone “*I benefit from other people using a cell phone while they drive (for example*, *they can call the ambulance and police if I am involved in a road traffic collision)”*.

*Support for legislation restricting cell phone use while driving*. The participants were also asked to indicate their agreement with the following two statements “*Talking on a cell phone is a matter of public safety; laws should be passed to restrict the usage of cell phones while driving*” and *“I oppose laws that limit the use of cell phones while driving”* to explore to what extend Iranian drivers support legislation restricting cell phone use while driving. They were also asked to choose the type of cell phone use they support being restricted, by selecting one of the following options: “*Handheld”*, “*Hands-free”*, “*In-vehicle system”*, “*All of the above”* and “*None of the above*”.

*Perceived risks of using a cell phone while driving*. The following three statements were used to evaluate participants’ perceived costs of using a cell phone while driving: “*Using a cell phone when I am driving threatens my personal safety and well-being*, *as well as other people*”, “*People using a cell phone while driving threatens my personal safety and well-being*”, and “*People using a cell phone while driving threatens the safety and well-being of others*”.

#### General personality traits

In the third section of the survey, the Persian adaptation [54] of the NEO-FFI [47] was administrated to investigate the following personality factors Neuroticism (N), Extraversion (E), Openness to Experience (O), Agreeableness (A), and Conscientiousness (C). Participants were asked to indicate their agreement with the 60 self-descriptive statements (12 statements per personality trait) using a 5-point Likert scale (1 = strongly disagree, 5 = strongly agree). Higher scores indicate higher levels of that particular trait.

### Data handling and analysis

All analyses were undertaken using SPSS v.24. Missing scale values, where there was a small number, were replaced using the mean of the participants’ answers for that scale. If a participant failed to answer several questions in a scale, the case was omitted. The data were categorised in the following way: age was categorized into three groups of < 30, 30–50 and > 50 years. Driving experience was divided into three categories of *low* (≤ 3 years), *medium* (< 10 years) and *high* (≥ 10 years) and in a similar manner time spent using a cell phone while driving each week was grouped into *low* (≤ 6 hours / week), *medium* (< 14 hours / week) and high (≥ 14 hours / week). The total frequency of CPWD (i.e. calling, texting and other cell phone features) per week was also calculated and stratified into five levels: *Never* (no use at all), *Rarely* (< 6 times / week), *Occasionally* (≤ 10 times / week), *Often* (≤ 20 times / week) and *Always* (> 20 times / week).

To account for the influence of driver characteristics on CPWD frequency, an ordered logit regression model was used with a log-log link function. Ordered discrete response models are derived by defining an unobserved variable that is employed as a basis for the ordinal ranking of data, which is specified as a linear function for each observation. Ordered logit/probit models have been previously used in different domains related to traffic safety, including the crash severity metric [55–61] and distracted driving [62, 63]. Moreover, both generalised ordered models and mixed generalized ordered models have been applied to allow the thresholds to change with the observed explanatory variables, unlike standard ordered logit and probit models [56]. A Bayesian spatial generalized ordered logit model is capable of considering the ordered nature and spatial correlation at the same time. For example, it has been previously used for examining freeway crash severity [56] and the impact of real-time weather conditions on this type of crash severity [57]. A comparison with the traditional generalised model also confirmed a better model fit for the spatial version [56]. In the driver distraction context, a multivariate ordered model in the Bayesian framework has been proposed to account for drivers’ decision to engage in risk-compensating behaviour [62].

Mixed logit models have also been employed to account for unobserved heterogeneity, which allows for the possibility that the impact of variables influencing crash frequency categories may change across observations and to relax irrelevant independence alternative (IIA) restrictions [64]. Analysing hourly crash likelihood of highway segments and examining the differences between single-and-multi vehicle accidents were some of their applications [65].

## Results

### Demographics and frequencies of cell phone use while driving

As shown in Table 1, only 7.1% of drivers reported never having used their cell phone in a week. In contrast, 32.5% reported always using their cell phone while driving. The participants had an average of 10.27 (*SD* = 3.19) years using a cell phone, with 66% reporting that the used hand-held cell phone while driving. Iranian drivers predominantly use their cell phones to make/receive calls (58.4%) and to use social networking applications (34.9%), as seen in Table 2. More than one third (36.1%) of the participants reported calling their parents while driving, followed by their spouse/fiancé (23.9%). The trend is somewhat different for texting behaviour: 28.6% of the participants stated that they were most likely to send/receive text messages to/from their spouse/fiancé, followed by friends (21.6%). Children were found to be the least frequent contacts in both tasks: 3.1% for both texting and calling tasks. More than 60% of the drivers supported legislation restricting handheld cell phone use and around 30% expressed their support for legislation prohibiting all modes of CPWD. Interestingly, only 5.5% of drivers did not support legislative bans. More than 70% of drivers reported that they had never used their cell phone while driving with a child passenger.

**Table 1:** Demographics and the total frequencies of a cell phone use.

Variable	Category	Frequency (%)	Total Frequency of a cell phone use (%)				
			Never	Rarely	Occasionally	Often	Always
Age	<30	150 (58.8)	7.3	22.7	20.0	16	34
	30–50	93 (36.5)	6.5	28.0	15.1	18.3	32.3
	>50	12 (4.7)	8.3	66.7	8.3	0	16.7
Gender	Male	169 (66.3)	5.9	23.7	18.9	14.2	37.3
	Female	86 (33.7)	9.3	32.6	15.1	19.8	23.3
Marital status	Single	136 (53.3)	3.7	22.8	17.6	18.4	37.5
	Married	119 (46.7)	10.9	31.1	17.6	13.4	26.9
Education	Below Diploma	11 (4.3)	0	27.3	18.2	18.2	36.4
	Diploma	58 (22.7)	5.2	29.3	13.8	19	32.8
	Undergraduate	145 (56.9)	7.6	27.6	17.2	13.8	33.8
	Postgraduate	41 (16.1)	9.8	19.5	24.4	19.5	26.8
Driving experience	Low	69 (27.1)	8.7	27.5	18.8	20.3	24.6
	Moderate	95 (37.3)	7.4	26.3	18.9	13.7	33.7
	High	91 (35.7)	5.5	26.4	15.4	15.4	37.4
Time spent driving per week	Low	68 (26.7)	16.2	39.7	17.6	13.2	13.2
	Moderate	91 (35.7)	4.4	26.4	18.7	16.5	34.1
	High	96 (37.6)	3.1	17.7	16.7	17.7	44.8
Total		255 (100)	7.1	26.7	17.6	16.1	32.5

**Table 2:** Types of use, peer-users and support for legislation by type of use.

Variable	Description/Category	Frequency (%)
Cell phone use Experience	M = 10.27, *SD* = 3.19	
Type of Cell Phone	Smartphone	242 (94.9)
	Non-smartphone	13 (5.1)
Mode of cell phone use while driving	Handheld	168 (65.9)
	Hands-free	31 (12.2)
	Bluetooth Headset	10 (3.9)
	In-vehicle system	28 (11)
Purpose of using a cell phone	Calling	149 (58.4)
	Texting	10 (3.9)
	Social networks	89 (34.9)
	Internet	7 (2.7)
Who do you call?	No calling	21 (8.2)
	Friends	33 (12.9)
	Parents	92 (36.1)
	Spouse/ Fiancé	61 (23.9)
	Children	8 (3.1)
	Colleagues	21 (8.2)
	Other	18 (7.1)
Who do you text?	No texting	32 (12.5)
	Friends	55 (21.6)
	Parents	42 (16.5)
	Spouse/ Fiancé	73 (28.6)
	Offspring	8 (3.1)
	Colleagues	14 (5.5)
	Other	29 (11.4)
Supporting legislation restricting:	Handheld	155 (60.8)
	Hands-free	7 (2.7)
	In-vehicle system	5 (2)
	All types of use	74 (29)
	None of the above	14 (5.5)
Using cell phone with a child passenger	Yes	73 (28.6)
	No	182 (71.4)

### Psychosocial variables and intercorrelations between variables

To explore the associations between the psychosocial variables and the reported frequencies of CPWD, a Mann–Whitney U test was conducted. As shown in Table 3, participants had higher self-perceived ability (M = 2.88) than others (M = 2.16), lower self-perceived risk (M = 3.24) than others (M = 3.39), more positive attitude towards their use (M = 1.78) than others use (M = 1.59) and higher self-perceived benefits from using cell phone while driving (M = 1.93) than for others (M = 1.84).

**Table 3:** The Mann–Whitney U test analysis of perceived ability, risks, support for legislation, attitudes and benefits between the two groups of users and non-users.

Psychosocial variables	Mean	*SD*	CPWD	Mean Rank	*U*	*Z*
Perceived ability (self)	2.88	1.19	No	71.61	1118*	-3.474
			Yes	132.28		
Perceived ability (others)	2.16	0.798	No	93.14	1505.5*	-2.286
			Yes	130.65		
Perceived risk (self)	3.24	0.765	No	191.94	982***	-4.212
			Yes	123.14		
Perceived risk (others)	3.39	0.635	No	174.53	1295.5**	-3.010
			Yes	124.47		
Support legislation	3.40	0.630	No	172.83	1326**	-2.844
			Yes	124.59		
Attitude (self)	1.78	0.620	No	89.53	1440.5*	-2.394
			Yes	130.92		
Attitude (others)	1.59	0.676	No	102.53	1674.5	-1.669
			Yes	129.93		
Perceived benefits (self)	1.93	0.800	No	89.93	1446*	-2.345
			Yes	130.90		
Perceived benefits (others)	1.84	0.776	No	113.94	1880	-.906
			Yes	129.07		

*Notes*.

*** Significant at *p* < 0.001

** *p* < 0 .01

* *p* < 0.05.

The Mann–Whitney U test also showed that the mean rank for perceived ability to drive safely while using a cell phone was significantly higher in distracted drivers than in non-users (132.28 vs. 71.61; *p* = 0.001). In addition, Table 3 also shows that distracted drivers reported a lower perceived risk of CPWD, both in the case of themselves (123.14 vs. 191.94, *p* < 0.001) and others (124.47 vs. 172.83, *p* = 0.003), a lower level of support for legal restrictions (124.59 vs. 172.83, *p* = 0.004), more positive attitude towards self-use (130.92 vs. 89.53, *p* = 0.017) and higher self-perceived benefits of CPWD (130.90 vs. 89.93, *p* = 0.015).

A Spearman’s rank order and Kruskal–Wallis tests, with the null hypothesis assuming the categories of use were identical within each of the psychosocial components, were conducted to investigate the association between reported frequency of CPWD and the psychosocial components. A level of 5% was considered significant. The results indicated a positive correlation between self-perceived ability and being a frequent user (0.309**). Table 4 shows that frequent users had a mean rank equal to 1.35 times higher than seldom users (χ^2^(4) = 31.21, *p* < 0.001). Furthermore, a significant negative correlation was found between total use and risk perception; i.e. the higher the perceived risk, both in the case of themselves (χ^2^(4) = 34.84, *p* < 0.001) and others (χ^2^(4) = 26, *p* < 0.001), the less they reported CPWD. A significant negative correlation (–0.215**) was also observed in support of legislation, wherein the frequency of CPWD decreased as support for the law increased (χ^2^(4) = 14.14, *p* = 0.007).

**Table 4:** Kruskal–Wallis and correlation tests for significant independent predictors of frequency of CPWD.

Psychosocial components	Level of use (CPWD Frequency)	Mean rank	χ2	Spearman’s rank correlation
Perceived ability (self)	Never	71.61	31.21***	0.309**
	Rarely	117.97		
	Occasionally	115.83		
	Often	118.12		
	Always	159.92		
Perceived ability (others)	Never	93.14	6.78	0.108
	Rarely	129.95		
	Occasionally	120.60		
	Often	132.06		
	Always	135.97		
Perceived risk (self)	Never	191.94	34.84***	-0.351**
	Rarely	147.55		
	Occasionally	128.46		
	Often	117.29		
	Always	103.16		
Perceived risk (others)	Never	174.53	26.00***	-0.308**
	Rarely	150.76		
	Occasionally	127.68		
	Often	112.44		
	Always	107.13		
Support for legislation	Never	172.83	14.14**	-0.215**
	Rarely	135.90		
	Occasionally	134.50		
	Often	123.02		
	Always	110.73		
Attitude (self)	Never	89.53	28.87***	0.333**
	Rarely	101.60		
	Occasionally	127.67		
	Often	130.15		
	Always	157.09		
Attitude (others)	Never	102.53	5.82	0.142*
	Rarely	121.74		
	Occasionally	123.17		
	Often	132.74		
	Always	138.93		
Perceived Benefits (self)	Never	89.83	44.39***	0.414**
	Rarely	97.75		
	Occasionally	114.97		
	Often	134.35		
	Always	164.99		
Perceived Benefits (others)	Never	113.94	6.67	0.152*
	Rarely	112.85		
	Occasionally	132.50		
	Often	131.52		
	Always	139.28		

*Notes*.

*** Significant at *p* < 0.001

** *p* < 0.01

* *p* < 0.05.

In addition, attitude towards self-use had a relatively moderate positive correlation with total use (0.333**), which became stronger as the frequency of CPWD increased (χ^2^(4) = 28.87, *p* < 0.001); this statement held true for the self-perceived benefits of CPWD, in which participants who reported CPWD most frequently had a mean rank nearly twice that of those who reported avoiding this behaviour completely (χ^2^(4) = 44.39, *p* < 0.001). Both attitudes towards others’ use and perceived benefits from others’ use showed positive correlations with total use (i.e. 0.142 and 0.152, *p* < 0.05), but differences among categories of use were not significant.

### Predicting the frequency of CPWD

Ordinal logistic regression, with a complementary log–log link function, was conducted on the aforementioned psychosocial variables, namely self-perceived ability, risk perception (both self and others combined), attitudes towards self and others’ use, support for legislation and the perceived benefits of CPWD. Driver-related factors including age, gender and driving experience were also entered into the model. Finally, the Big Five personality traits were also entered into the model. The mean scores for the Big Five personality traits, i.e. openness to experience, conscientiousness, extraversion, agreeableness and neuroticism were reported as 25.54, 33.37, 28.4, 28.5 and 20.69, respectively. The formulation of the model is as follows:

$$z_{i}=\beta X_{i}+\varepsilon_{i}$$ (2)

where *i* (*i* = 1,2,………,*N*) represents the individual, *z*_*i*_ is a linear function of covariates *X*_*i*_, *β* is a vector of associated estimable parameters with *X*_*i*_ and *ε*_*i*_ is a residual term with a logistic distribution (random disturbance). *y*_*i*_ which is the observed ordinal data can be defined for each observation as follows:

$$y_{i}=\{\begin{array}{l} 1\;\mathrm{if}\;z_{i}\leq \mu_{0}\\ 2\;\mathrm{if}\;\mu_{0}<z_{i}\leq \mu_{1}\\ 3\;\mathrm{if}\;\mu_{1}<z_{i}\leq \mu_{2}\\ \ldots \\ J\;\mathrm{if}\;Z_{i}\geq \mu_{J-2\prime } \end{array}$$ (3)

where *μ* are estimable parameters (or thresholds that are estimated jointly with *β*) that define *y*, which corresponds to the orderings on integers and j (j = 1, 2,……, J) is the usage level in which J is the highest integer ordered response. Here, qualitative frequencies are converted to integers without loss of generality. In order to guarantee the well-defined intervals and natural ordering of observed severity, the thresholds are considered to be ascending in order, such that *μ*_0_<*z*_*i*_ < ……… *μ*_*J*_ where *μ*_0_ = -∞ and *μ*_*J*_ = +∞. The probability expressions take the following form:

$$\pi_{ij}=\mathrm{Pr}\left(y_{i}=j|X_{i}\right)=\Lambda \left(\mu_{j}-X_{i}\beta \right)-\Lambda \left(\mu_{j-1}-X_{i}\beta \right)$$ (4)

where *Λ*(∙) is the standard logistic cumulative distribution function and *π*_*ij*_ is the probability that individual *i* keeps a usage level *j*. As *Λ*(*t*) in Eq (4) equals $\frac{1}{1+e^{-t}}$, the probability takes the following form:

$$\pi_{ij}=\mathrm{Pr}\left(y_{i}=j|X_{i}\right)=\frac{exp\left(\mu_{j}-X_{i}\beta \right)}{\left(1+exp\left(\mu_{j}-X_{i}\beta \right)\right)}-\frac{exp\left(\mu_{j-1}-X_{i}\beta \right)}{\left(1+exp\left(\mu_{j-1}-X_{i}\beta \right)\right)}$$ (5)

The parameter estimates are calculated by the log-likelihood estimate. For a population of N participants, the likelihood function for the ordered logit model is as follows:

$$LL=\sum_{i=1}^{N}\sum_{j=1}^{J}\delta_{ji}LN\left[\Lambda \left(\mu_{j}-X_{i}\beta \right)-\Lambda \left(\mu_{j-1}-X_{i}\beta \right)\right]$$ (6)

where *δ*_*ji*_ equals to 1 if the observed outcome is j and 0 otherwise.

Table 5 shows the result of the logistic regression, with all variables being entered at the same time. The table shows that for a one-unit increase in self-perceived ability, holding the other variables in the model constant, the log-odds of CPWD frequency would increase by 17.9%. Likewise, a one-unit change in self-perceived benefits increased the log-odds of CPWD by 56.7%. Conversely, a one-unit increase in risk perception would diminish the log-odds of CPWD by 25.8%. Moreover, CPWD in the presence of a child had a prominent impact on the frequency of use, where a positive change would result in an 85.1% increase in the odds of being in the ‘always’ category. In driver-related factors, only age was significant and moving from >50 age category to 30–50 and <30 years categories resulted in increasing the odds of total use by 3.41 and 3.23, respectively.

**Table 5:** Factors related to the frequency of CPWD.

Variable		Estimate	Std. Error	Wald	95% Confidence Interval	
Perceived ability (self)		0.179	0.76	5.482*	0.029	0.328
Risk perception		-0.258	0.078	13.549***	-0.437	-0.133
Attitude (self)		-0.116	0.195	0.354	-0.497	0.266
Attitude (others)		-0.012	0.152	0.007	-0.310	0.258
Support legislation		0.166	0.149	1.242	-0.126	0.457
Perceived benefits (self)		0.567	0.148	14.612***	0.276	0.857
Perceived benefits (others)		-0.163	0.111	2.156	-0.380	0.055
CPWD in presence of a child		0.851	0.224	14.494***	0.413	1.289
Age	<30	1.173	0.409	8.221**	0.371	1.975
	30–50	1.228	0.375	10.730**	0.493	1.963
Gender	Male	-0.105	0.183	0.330	-0.464	0.254
Driving experience	Low	-0.350	0.282	1.543	-0.902	0.202
	Medium	-0.383	0.246	2.426	-0.865	0.099
Big Five	Agreeableness	-0.037	0.018	4.174*	-0.073	-0.002
	Conscientiousness	-0.035	0.015	5.150*	-0.064	-0.005
	Extraversion	0.055	0.018	9.139**	0.019	0.090
	Neuroticism	-0.03	0.013	0.192	-0.031	0.020
	Openness to experience	-0.01	0.017	0.004	-0.034	0.032
Log likelihood (Final) 658.475						
Nagelkerke Pseudo R^2^ 0.366 Cox and Snell Pseudo R^2^ 0.348						

*Notes*.

*** Significant at *p* < 0.001

** *p* < 0.01

* *p* < 0.05.

As for the Big Five personality traits, agreeableness and conscientiousness showed a negative relationship with frequency of use. A one-unit increase in agreeableness resulted in log-odds of CPWD being decreased by 0.037 while for conscientiousness a one unit increase in conscientiousness was associated with a decline (0.035) in the log-odds of CPWD. Finally, extraversion had a significant Wald statistic, with a one unit increase in extraversion resulting in a 5% increase in CPWD use.

## Discussion

In Iran, like in many other countries, using a cell phone while driving is legally prohibited, due to its negative impact on driving performance which increases the risk of RTAs. Therefore, the aim of this study was to explore the self-reported frequency of cell phone use while driving in a sample of the Iranian drivers. In addition, we explored the associations self-reported cell phone use had with several psychosocial factors and the Big Five personality factors. Overall, our study found that an alarmingly large proportion of Iranian drivers regularly use their handheld cell phones while driving. Interestingly, this issue remains understudied, despite the substantial implications of this behaviour for traffic safety.

Iranian drivers reported using their cell phones mostly for calling, followed by using social networking applications and sending/receiving text messages. Surfing the Internet was the least commonly reported behaviour amongst participants. This is somewhat consistent with previous research which reported that drivers most frequently use their cell phones for answering/making calls, with much smaller proportions of drivers reporting reading/sending text messages while driving [66, 67]. The tendency of drivers to use cell phones for calling, as opposed to texting, can be explained in terms of the cognitive and visual demands that the reading/writing task poses to drivers’, which makes this behaviour more challenging to perform while driving [11, 68]. Our findings also indicate that drivers regularly use various cell phone application, like social networking applications and the Internet. This finding is in line with the results of study conducted by the Royal Automobile Club [69] which reported that one quarter of young drivers use social networking applications while driving. Given that only a small body of research has explored the effect of using various popular smart phone applications on driving performance, future research is needed to investigate this important issue.

Our findings also showed that participants would predominantly call their parents and partners while driving. As for texting, a slightly different pattern was observed, as mostly they texted their partners and friends. This suggests that significant others may have a major influence on drivers’ CPWD behaviour, which aligns with previous research indicating that drivers use their cell phones more frequently when their significant others approve of this behaviour [41, 70]. In addition, Iran is one of the countries where family ties play a central role in people’s every-day lives [71], which suggests that it is important to incorporate themes of social influence when developing interventions to tackle CPWD in Iran.

Despite the prevalence of cell phone use amongst the participants, our results also showed that more than half of them supported a legislative ban of handheld cell phone use while driving, and over one quarter supported legislative bans for both handheld and hands-free cell phone use. These results clearly suggest that drivers perceive using a handheld cell phone as riskier than using a hands-free device, which is in line with research conducted in New Zealand [72] and the United Kingdom [45]. As for the inconsistency found in the reported behaviours, i.e. supporting legislation and still engaging with CPWD, Sanbonmatsu et al. [33] suggested this to be considered as hypocrisy, which is quite common amongst drivers.

Interestingly, most drivers reported that they would never use their cell phones while driving if there was a child passenger in their vehicle, which could therefore be considered as one of the protective factors against this behaviour. This could be explained in terms of the driver-passenger emotional relationship, as suggested by Megias and colleagues [73] who found that drivers perceived a higher level of risk when the passengers were their significant others (in particular children), as opposed to work colleagues. This finding should also be considered when designing campaigns against CPWD behaviour.

The study findings indicated that the participants assess the risks associated with CPWD as larger when it comes to other drivers engaging in this behaviour. They evaluated their own ability to drive safely and use a cell phone as being better than other drivers’ and believed that they can benefit more from CPWD with lower risks, in comparison to other drivers. As previously mentioned, scholars explain this tendency in terms of drivers’ overconfidence and suggest that drivers tend to believe that they are more skilful than others [74–76]. Our results also showed that participants overall had more negative attitudes toward other drivers using a cell phone. This finding is rather alarming, as previous research found that overconfidence is strongly associated with more risk-taking behaviours [76–78]. In addition, McKenna and Horswill [79] and Martinussen and colleagues [80] reported that overconfidence can also lead to unsafe driving behaviours. Drivers, who never use their cell phones while driving also believed that they were less able to drive safely and use a cell phone, and that there are more risks and less benefits associated with this behaviour, in comparison to those drivers who frequently use a cell phone while driving.

The results of the correlational analysis showed that overall those drivers who use their cell phones frequently believed that they are more able to drive safely and use a cell phone. In addition, those drivers who had higher risk perceptions use their cell phones less frequently. A negative association was also found between support for legislation and the frequency of a cell phone use, suggesting that those drivers who support the legislative bans are also those that use their cell phones less frequently while driving. Lastly, those drivers who had positive attitudes towards CPWD and believed that this behaviour could be beneficial, also used their cell-phones more frequently.

Finally, the results of the logistic analysis showed that the perceived ability and benefits of CPWD, lower risk perceptions, and younger age, were significantly and positively associated with frequent cell phone use. In terms of personality traits, agreeableness and conscientiousness were found to be negatively associated with frequency of CPWD, which is consistent with previous research, which reported that people exhibiting these personality traits were less prone to engage in risky behaviours [81]. On the other hand, extraversion had a positive relationship with CPWD. Thompson [82] suggests that extraverted people are generally more talkative and enthusiastic, which means they are more interested in connecting with others. This finding was consistent with Parr et al. [51] and Braitman and Braitman [52], who also found that extraversion was strongly associated with drivers’ intentions to multitask.

## Study limitations

This study still has several limitations. Firstly, the study utilised a self-reported questionnaire, meaning that responses could have been affected by social desirability bias. To mitigate the effect of this phenomenon, as suggested by previous research [83], participants were assured that their responses would be treated confidentially and anonymously. Secondly, this sample of Iranian drivers is unlikely to be representative of all drivers in this country, as Iran has an underdeveloped infrastructure and a heterogeneous population in terms of socioeconomic status and lack of police enforcement [84]. Unfortunately, the present study did not have the resources to fund an alternative method of data collection. Lastly, most of the participants were recruited from the universities and other organisations, which means that they potentially belong to higher socioeconomic groups and have different perceptions of road safety than the rest of the population. Considering these points, it is likely that this sample of Iranian drivers was not representative of the Iranian population of drivers. Future research should therefore explore the self-reported frequencies of CPWD and its underlying psychosocial factors using a more representative sample of the Iranian drivers.

## Conclusions and practical implications

In conclusion, our study found that the vast majority of the drivers regularly use their cell phones while driving. Overall, psychosocial variables, such as age, presence of a child, perceived benefits and risks associated with CPWD, as well as perceived ability to drive safely while using a cell phone, were strongly related to self-reported frequency of cell phone use. As for personality traits, extraversion, agreeableness and conscientiousness were also found to be significantly associated with the reported frequency of CPWD.

Considering the fact that the functionality of smart phones is growing, and more applications are being introduced, it is possible that CPWD may increase significantly in the future. The results of this study suggest that targeted educational campaigns should be developed incorporating a psychosocial profile of drivers who are prone to engage in this behaviour.

## Supporting information

S1 Data (XLSX)

S2 Data (DOCX)
