# Supplementary material for: Psychosocial factors associated with the self-reported frequency of cell phone use while driving in Iran
Source: PLoS One. 2021 Apr 21;16(4):e0249827. doi: 10.1371/journal.pone.0249827 (PMC8059850; doi:10.1371/journal.pone.0249827)
Supplement: S2 Data — (DOCX) [file pone.0249827.s002.docx]

**Highlights**

- Drivers’ demographic characteristics, thoughts and opinions, personality traits, and CPWD frequency were assessed.
- An alarming number (93%) of the drivers in the study were using their cellphones while driving at least one time a week.
- Hypocrisy and overconfidence were prominent among drivers.
- Drivers’ thoughts and opinions about multitasking both in terms of themselves and others played a crucial role in CPWD frequency.
- Next to raising awareness campaigns, focus should be shifted into other facets of distracted driving like education regarding overconfidence and also legislation should incorporate other factors such as personality traits in the process of obtaining driver’s licence.
